# BUL: a novel lectin from *Bauhinia ungulata*L. seeds with fungistatic and antiproliferative activities

**DOI:** 10.1186/1753-6561-8-S4-P87

**Published:** 2014-10-01

**Authors:** André Silva, Talita Leite, Edson Teixeira, Luiz Ponte, Luciano Pinto

**Affiliations:** 1 Universidade Federal do Ceará, Fortaleza, Brazil; 2 Universidade Estadual do Vale do Acaraú, Sobral, Brazil; 3 Universidade Federal de Pelotas, Capão do Leão, Brazil

## 

A new galactose-binding lectin, termed BUL, has been purified from seeds of *Bauhinia ungulata *(Caesalpinoideae) by precipitation with solid ammonium sulfate followed by agarose-lactose affinity chromatography. *B. ungulata *lectin strongly agglutinated rabbit erythrocytes, both native and treated with proteolytic enzymes, and was inhibited by D-galactose and D-galactose-derived sugars, especially N-acetyl-D-galactosamine. BUL was shown to be a stable glycoprotein, maintaining its hemagglutinating activity after incubation at wide ranges of temperature and pH, but not after incubation with EDTA. By SDS-PAGE analysis under reduced conditions, purified BUL showed an electrophoretic profile consisting of a single band with apparent molecular mass of 30 kDa. BUL showed intrinsic fluorescence typical of folded globular proteins, and circular dichroism spectra of lectin in the native state showed a predominance of β-sheet secondary structure. The N-terminal amino acid sequence of 19 residues showed a high sequential similarity to other galactose-specific lectins from the *Bauhinia *genus. In addition, BUL showed antifungal activity against phytopathogenic species and showed *in vitro *antiproliferative activity against the HT-29 cell line of human colon adenocarcinoma in a dose-dependent manner.

